# The role of booster vaccination and ongoing viral evolution in seasonal circulation of SARS-CoV-2

**DOI:** 10.1098/rsif.2022.0477

**Published:** 2022-09-07

**Authors:** A. N. M. Kraay, M. E. Gallagher, Y. Ge, P. Han, J. M. Baker, K. Koelle, A. Handel, B. A. Lopman

**Affiliations:** ^1^ Department of Kinesiology and Community Health, University of Illinois at Urbana-Champaign, Urbana, IL, USA; ^2^ Institute for Genomic Biology, University of Illinois at Urbana-Champaign, Urbana, IL, USA; ^3^ Johns Hopkins University Applied Physics Laboratory, Laurel, MD, USA; ^4^ School of Health Professions – Public Health, University of Southern Mississippi, Hattiesburg, MS, USA; ^5^ Rollins School of Public Health, Emory University, Atlanta, GA, USA; ^6^ Department of Biology, Emory University, Atlanta, GA, USA; ^7^ Department of Epidemiology & Biostatistics, University of Georgia, Athens, GA, USA; ^8^ Center for the Ecology of Infectious Diseases, University of Georgia, Athens, GA, USA

**Keywords:** booster, evolution, vaccination, COVID-19, non-pharmaceutical interventions

## Abstract

Periodic resurgences of COVID-19 in the coming years can be expected, while public health interventions may be able to reduce their intensity. We used a transmission model to assess how the use of booster doses and non-pharmaceutical interventions (NPIs) amid ongoing pathogen evolution might influence future transmission waves. We find that incidence is likely to increase as NPIs relax, with a second seasonally driven surge expected in autumn 2022. However, booster doses can greatly reduce the intensity of both waves and reduce cumulative deaths by 20% between 7 January 2022 and 7 January 2023. Reintroducing NPIs during the autumn as incidence begins to increase again could also be impactful. Combining boosters and NPIs results in a 30% decrease in cumulative deaths, with potential for greater impacts if variant-adapted boosters are used. Reintroducing these NPIs in autumn 2022 as transmission rates increase provides similar benefits to sustaining NPIs indefinitely (307 000 deaths with indefinite NPIs and boosters compared with 304 000 deaths with transient NPIs and boosters). If novel variants with increased transmissibility or immune escape emerge, deaths will be higher, but vaccination and NPIs are expected to remain effective tools to decrease both cumulative and peak health system burden, providing proportionally similar relative impacts.

## 1. Introduction

COVID-19 has caused catastrophic loss of life and health system strain in the United States [1]. Since the US epidemic first began in early 2020, multiple transmission waves have occurred, triggered by changes in social distancing and circulating variants. In response, the US government enacted widespread non-pharmaceutical interventions (NPIs) to slow epidemic spread. Over time, these measures relaxed in favour of more targeted approaches, particularly as vaccination became more available.

As of June 2022, three types of highly effective COVID-19 vaccines are available in the USA [2], two of which have been authorized for emergency use in children six months and above [3]. However, vaccine effectiveness has decreased over time, partly due to waning efficacy and partly due to the emergence of novel variants against which vaccines are less effective [4]. As a result, booster doses have been recommended to maintain efficacy [2].

The US epidemic beginning in early 2020 was triggered by the Wuhan variant, with the winter 2020 surge driven by the Delta variant, which was more infectious and severe than prior dominant variants [5,6]. In late 2021, the Omicron variant began to predominate. The Omicron variant seems to be more transmissible but less severe than prior variants [6,7]. Moreover, vaccine efficacy against both infection and severe disease appears to be lower against the Omicron variant [8]. However, booster doses may at least temporarily increase vaccine efficacy against circulating variants [9].

As circulation continues, ongoing SARS-CoV-2 evolution might allow immune escape to occur, influencing the intensity of follow-up waves [10]. Additional NPIs might be rolled out in response to subsequent surges in cases, influencing the severity of future transmission waves. We used a transmission model to evaluate how booster doses and resuming NPIs amid ongoing pathogen evolution might influence the severity of SARS-CoV-2 transmission.

## 2. Methods

**Figure 1.**
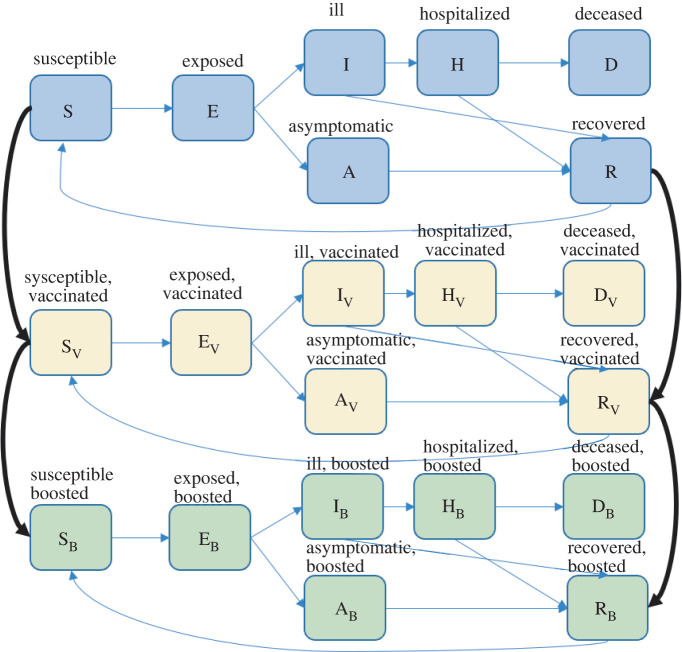
Transmission model. Individuals are classified as susceptible (S), latently infected (E), symptomatic (I), asymptomatic (A), hospitalized (H), recovered (R) or deceased (D). Individuals are also classified by their vaccination status as either unvaccinated (blue), vaccinated with two doses (yellow), or vaccinated and boosted (green). Individuals who are infected with SARS-CoV-2 wane into their own infection class.

Our transmission model extends classic compartmental susceptible–exposed–infected–recovered (SEIR) epidemiological models to incorporate additional features relevant to the transmission dynamics of SARS-CoV-2. Our SEIR-like model includes seven compartments (figure 1). Initially, many individuals are susceptible to infection (S). Upon exposure, they enter a latent period (E), during which they cannot transmit. They can then develop asymptomatic infection (entering the A class) or symptomatic infection (entering the I class). We assume that all asymptomatic individuals will recover (R). Those with symptomatic infections can either recover (entering the R class) or require hospitalization (entering the H class). Some of those hospitalized will die (entering the deceased class D), and the rest will recover (entering the R class). After immunity wanes, individuals in the R class return to their S class, depending on vaccine status. The model was also stratified by age (less than 20 years, 20–64 years and greater than or equal to 65 years) and risk of acquiring infection (high versus low).

Vaccination was implemented by adding a daily rate of vaccination and additional compartments for vaccinated individuals, which mirror the compartments in the base model: S_V_, E_V_, A_V_, I_V_, R_V_, H_V_ and D_V_. For simplicity, we assume that individuals in the S or R classes can be vaccinated but individuals in the other compartments will not be. Following vaccination, susceptible individuals S move into the susceptible vaccinated compartment S_V_, and all recovered individuals move into the recovered vaccinated compartment R_V_. We used coverage estimates from 6 January 2022 to estimate the baseline prevalence of vaccination [1]. We assume that all adults who intend to be vaccinated have already done so and that future vaccines will be allocated to children or will be booster doses, with peak child vaccine coverage being reached by late October 2022, consistent with current vaccine rates [1]. Baseline immunity and initial age distribution of infections were estimated using seroprevalence data [11]. Primary series vaccination was modelled as reducing the risk of severe disease by 70% (50% reduction in infection and 40% reduction in hospitalization given infection) [12] and 90% if booster doses are used (50% reduction in infection and 80% reduction in severe disease given infection) [9]. In the absence of any new vaccine products, we assume that all boosted individuals remain current on their boosted status and that any further vaccinations do not increase efficacy further such that only one boosted class is sufficient.

We also consider potential impacts if a variant-specific booster becomes available in autumn 2022 (electronic supplementary material, figure S2). For this analysis, we added a third vaccine class to the model, with vaccine efficacy against infection being increased to 90% for individuals with the variant-adapted booster, consistent with initial efficacy data for both mRNA vaccines [13]. Vaccine efficacy against severe disease was assumed to be similar with first and second boosters, such that the combined effectiveness against hospitalization with an adapted booster was 98%. All booster doses administered after 1 September, 2022 were assumed to be with the variant-adapted booster.

To parametrize this model, we set parameter values where known. For uncertain parameters, we selected model parameters based on both reasonable values from the literature and the ability of the model to reproduce Omicron dynamics. Model parameters are shown in the electronic supplementary material, table S1. As the duration of immunity is uncertain, we also conducted a sensitivity analysis with a shorter duration of immunity (90 days).

We assume that relaxation of NPIs began in March 2022, reaching baseline [14] (i.e. pre-pandemic) levels by 7 July 2022. This timeline is consistent with announced policy decisions in multiple US states [15]. We consider a scenario where these NPIs remained at their relaxed level as the seasonal transmission rate increases. We then explore whether a temporary increase in social distancing to levels observed during Omicron might impact risk if implemented in autumn 2022.

To explore impacts in the context of ongoing pathogen evolution, we first use model parameters consistent with the circulating Omicron variant (electronic supplementary material, table S1) for 1 year, beginning 7 January 2022. Our seasonal transmission model implies that transmission will increase in the autumn, even if no novel variants emerge. We then consider how increasing transmissibility might impact model dynamics. For this scenario, we assume that the novel variant begins to emerge in the autumn, coincident with increases in the seasonal transmission rate and that the maximum seasonal beta term is increased by 20–50% at the start of the autumn. The circulation of this variant thus has the potential to drive a surge in infections beyond the effect that increases in the seasonal transmission rate can have on driving an autumn wave. We also consider how changes in the new variant's ability to escape immunity might impact dynamics by increasing the rate of waning immunity by 50%, consistent with immune escape.

## 3. Results

**Figure 2.**
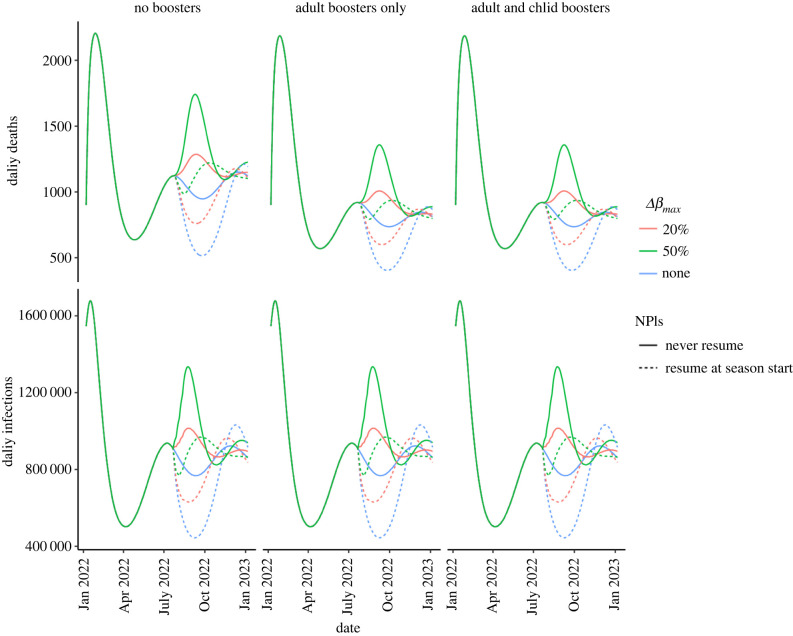
Daily deaths (top row) and infections (bottom row) from SARS-CoV-2 waves as a function of immune escape, booster vaccine strategy and social distancing.

Our model closely matches peak daily deaths and hospitalization rates observed for the Omicron wave in January and February 2022, with peak daily deaths around 2200 day^−1^. In our model, incidence is predicted to rebound in June 2022 as NPIs relax and natural immunity wanes (figure 2). This rebound is expected to be largely driven by relaxing NPIs, with waning immunity playing a secondary role. Specifically, deaths were predicted to increase from a low of 520 to 1064 day^−1^ after 180 days (a 104% increase). Twenty-five per cent of this increase was attributable to waning immunity alone, with the remainder being due to relaxing NPIs and the interaction between NPI relaxation and waning immunity (electronic supplementary material, figure S1 and text). Booster doses can influence the intensity of this spring/summer resurgence. Daily deaths at the end of the summer wave (on 21 July 2022) were 18% lower if boosters were used.

Additionally, seasonal changes in transmission are expected to produce a new transmission wave in autumn 2022. The severity of this autumn transmission wave will depend on the implementation and uptake of public health interventions over the coming months as well as ongoing variant evolution. In the absence of a new immune variant emerging that has increased transmissibility, high uptake of booster doses could reduce cumulative deaths between March 2022 and January 2023 by 20% while also reducing the intensity of the autumn surge. Reintroducing NPIs as the seasonal transmission rate begins to increase can also further reduce risk and could effectively halt an autumn resurgence if immune escape does not occur or if its level is low (figure 2). Combining booster doses with NPIs could reduce cumulative deaths by up to 30% compared with a scenario without booster doses or NPIs. Increased transmissibility could increase peak deaths, but the per cent reduction in deaths achievable through booster vaccination is similar (the fraction of preventable deaths is 20% with 50% immune escape and increases to 27% with combined boosters and NPIs). Similarly, immune escape could lead to increased deaths (electronic supplementary material, figure S4), but booster vaccination remains beneficial. Roll-out of variant-specific boosters that have increased effectiveness against infection could be beneficial, but predicted impacts are small due to the expected constant roll-out rates of these variant-specific boosters (e.g. we do not model an aggressive vaccine campaign in autumn 2022). Specifically, if variant-adapted booster vaccination continues at the roll-out rates observed in January 2022, combined boosting and NPIs could reduce deaths by 34% without any increases in transmissibility or 30% with increased transmissibility (electronic supplementary material, table S3).

In addition to reducing deaths, NPIs also reduced overall infections (figure 2). Without immune escape, NPIs alone could reduce infections by 16% between 21 July 2022 and 6 January 2023. If immune escape occurs, the overall impact of NPIs is reduced, with similar NPIs reducing infections by 11% during the autumn. By contrast, standard booster doses do not impact overall infection rates. Adapted booster doses could reduce infection rates in future waves, but are unlikely to do so substantially during the autumn 2022 wave. We also found that the benefit of NPIs is specific to high transmission periods—when booster vaccines are used, allowing NPIs to relax during the summer and reintroducing in the autumn provides similar benefits to indefinite NPIs (307 000 deaths with indefinite NPIs compared with 317 000 with targeted NPIs).

While reintroducing NPIs during the autumn was beneficial, our model also revealed that relaxing NPIs during the summer was beneficial for follow-up transmission waves because it allowed for population immunity to accrue, reducing the number of people who were simultaneously susceptible to infection. When NPIs are sustained, the overall number of susceptible individuals is about 30% higher at the start of the autumn surge compared with the scenario where NPIs are allowed to relax. For this reason, daily deaths are higher if a novel variant with increased transmissibility emerges and NPIs are sustained indefinitely (electronic supplementary material, figure S1).

As a sensitivity analysis, we considered implications if natural infection provides additional immunity equivalent to receiving either (i) a two-dose series of vaccine or (ii) a booster dose (electronic supplementary material, figure S3). In this sensitivity analysis, the patterns seen in the overall results were similar, but deaths and infections were lower. For example, combining NPIs and booster doses reduced deaths by 25% without immune escape for the hybrid immunity extension compared with 30% in the base model. However, overall deaths between January 2022 and January 2023 were 237 000 with hybrid immunity, boosters and NPIs compared with 317 000 for the same scenario without hybrid immunity (electronic supplementary material, table S3 and figure S3).

We also considered implications if the duration of immunity to COVID-19 lasted three months rather than the seven months we used for our main model simulations. While peak deaths and were higher in this model and the autumn surge was more intense, the impact of boosting and reintroducing NPIs was similar (electronic supplementary material, figure S6).

## 4. Discussion

Our model predicts both a mid-year peak (driven by relaxing NPIs) and an autumn resurgence (driven by seasonal increases in the transmission rate). However, public health interventions can substantially impact these dynamics; combining boosters and NPIs could reduce deaths by up to 34%. If a novel variant emerges that escapes immunity, deaths could increase, but both interventions remain beneficial. While combining booster doses and NPIs reduce morbidity and mortality, we find that targeting NPI use to high transmission periods has similar benefits to indefinite NPIs. Thus, widespread use of booster doses can allow NPIs to remain more relaxed with relative safety.

While vaccines may not be as effective against infection with novel variants, evidence to date suggests that existing vaccines are likely to provide good protection against more severe disease, even for genetically distant variants like Omicron [16], particularly if booster doses are used [17]. New vaccine formulations targeted to circulating variants have the potential to improve efficacy. However, in our model, the impact of adapted boosting was modest. This pattern probably occurred because booster doses were not administered quickly enough to curtail an autumn transmission surge. We predicted that 30% of older adults, 27% of adults and 17% of children would be vaccinated with a variant-adapted booster by January 2023. Faster roll-out rates might enhance impacts beyond what we have modelled.

These findings are meant to be understood qualitatively in terms of the overall pattern of risk rather than predicting a precise level of future burden; real disease risk is likely to be lower than we have modelled for a few reasons. For example, while we predict a relatively high level of ongoing deaths, fatality from COVID-19 may be lower due to both incomplete waning of immunity and improved treatment. We have used simple assumptions in this model, with an average immunity level of seven months followed by complete waning to baseline risk by vaccine group. However, immunity may accrue more gradually over repeated infections and reach a higher level, as has been shown for other pathogens [18] and preliminary data show might also be true for SARS-CoV-2 [19]. Our sensitivity analysis suggests that this tendency would probably decrease deaths, but would not impact the overall utility of booster doses and NPIs.

In conclusion, our simulations suggest that resurgences in COVID-19 cases are likely both in the summer, as NPIs continue to relax, and in the autumn, as seasonal factors push the transmission rate higher. However, widespread use of booster doses can make a substantial impact on the ongoing public health burden, even as novel variants continue to emerge. Re-establishing NPIs can also reduce the impact of future transmission waves. While ongoing viral evolution that results in increased transmissibility may increase cases and deaths, both vaccines and NPIs remain beneficial to reduce the public health burden of future surges of COVID-19.

## Data Availability

All relevant data have been previously published and are cited appropriately or are contained within the article and its electronic supplementary material [20].
